# Extra Supplement—The Nursing Mirror

**Published:** 1889-06-29

**Authors:** 


The Nursing Mirror.

All Contributions for this Supplement should be addressed "The Nursing Mirror," care of the Editor, 139-140, Salisbury Court, Fleet Street, London, E.C.

### En Passant.

MARYLEBONE INFIRMARY.—Miss Vincent reports that nine probationers were trained here last year. The lectures given by the medical superintendent, Mr. J. R. Lunn, and the weekly classes held by the assistant matron, Miss Moriarty, had been well attended. By agreement with the guardians, the council have increased the contribution to this infirmary, so as to enable the number of probationers to be increased to fifteen.

DUBLIN NURSING INSTITUTION.—Lady Londonderry lately visited this institution and presented twenty-six nurses with prizes and certificates gained. There are sixty-seven nurses in training, and the directors of the institution have undertaken the nursing of several hospitals outside Dublin. The fees received for nursing and from probationers were last year £1,746 4s. 3d., and the profit, £384 3s.; out of this the directors distributed £135 as good service rewards, varying in amount from £9 to £3, according to length of service.

THE NIGHTINGALE SCHOOL.—The report for 1888 has just been issued, and states that from the opening of the school in June, 1860, to the end of 1888, a total of 965 candidates have been admitted, and 578 have, after completing a year's training, received appointments in some public hospital or infirmary, or other institution for the benefit of the sick poor. The twenty-nine probationers who completed their training received the following appointments—viz: St. Thomas's Hospital, one ward sister, assistant night superintendent, one night nurse, three day nurses, and fifteen extra nurses; Metropolitan and National Nursing Association, Bloomsbury-square, two nurses: General Hospital, Cheltenham, two ward sisters; and St. Marylebone Infirmary, three day nurses. The lectures have been delivered as usual, and the council express their thanks to the lecturers, and to the matron, Miss Pringle.

A COLONIAL NURSE.—The *Illustrated Sydney News* for March 7 contained an account of the Glebe Hospital for Children, Sydney, Australia. There were some good illustrations of the wards, where the transparent mosquito curtains to every bed looked strange, and a portrait of Miss Maud Lobb, the well-beloved matron, whose appointment we chronicled last year. This is what the reporter says of her: "As I glance at the fair-haired young hospital matron, in her straight black dress, snowy cap and apron, the lot of a nursing sister seems simple and poetic enough—a life secluded from the world, a little child claiming caressing love." And as he is going away, this is what the nurses say of her: "But they want more than this—the sympathy which lightens work. They receive it in abundance from their beloved head. 'Say a good word for our matron,' pleaded one sunny-faced, gentle-fingered young nurse; 'she is a matron in a thousand, so considerate to her nurses.'" Miss Lobb's remarks on hip-disease are worth repeating: "'Why is there so much hip-disease here?' I ask. 'The old world reason so often given, "carrying mother's baby," does not hold good in Australia.' 'Bad blood,' says the matron wearily; 'accidents, an exhausting climate, and, oftener still, unwholesome feeding and superfluous meat diet.'"

THE PENSION FUND.—After careful consideration the committee of the Royal Hants County Hospital have determined to avail themselves of the advantages of the National Pension Fund for the benefit of their nurses. The matter having been carefully thought out by a special committee, was brought before the general committee on the 5th inst., and they agreed that so long as the financial state of the hospital would allow it, they would pay one-half of the premiums of their nurses out of the nursing fund. It is believed that the nurses of the hospital, who have attained a high standard of excellence, will largely take advantage of this well-deserved boon on the part of the committee, who are to be most highly commended for the action they have taken. An announcement with regard to the success of the fund may shortly be expected.

SHORT ITEMS.—Dr. Sheen, medical superintendent of the Cardiff workhouse, has inaugurated trained nursing in the wards of the infirmary.—The *Medical Press* says: "We have long urged that the registration of midwives was indispensable, if only to effectually shut out the successors to Mrs. Gamp, but sick nurses are usually obtained from institutions which only send out properly qualified women." The *Dispatch* says with regard to registration: "The nurse of the future is to have her Royal College, like the physician and the surgeon. Philanthropic snobbery, with all its vulgar wealth and cant, has been enlisted in support of a movement to raise the status of this useful and self-sacrificing class of women. Where will the interests of the poor come in in all this movement for elevating the nurse's vocation to the status of a profession? Are nurses' fees to be raised to the level of doctors' fees! Does the good Princess intend to tack on to her movement an institution that will provide registered nurses for the poor on terms within their frugal means?"

REGISTRATION.—A correspondence on this subject has been going on in the *St. James's Gazette*, and we quote the following few lines from the letter of "A Hospital Worker": "Though the Royal charter were granted and registration made compulsory, the British Nurses' Association would be powerless to institute examinations, to put a nurse's name on the register, or to erase it from it. I regret that the 'Hospital Physician' cannot write a letter without asking for money. I am not aware that the public has largely supplied the British Nurses' Association with the 'sinews of war'; though I am well aware that the association has issued a circular to its members asking them to beg from their friends and even from their patients—a thing which has hitherto been considered dishonourable by all self-respecting nurses, and which is forbidden by the best institutions." The *Manchester Guardian* has also had a letter on the subject by a "Hospital Superintendent," who says: "Whilst avoiding anything approaching to personal controversy, the question is so important to our nurses that it is necessary that the public as well as the nurses should fully realise that the most experienced of the hospital managers, with the heads of the nurses' training schools, from the Nightingale Fund downwards, are firmly convinced that were the scheme of registration proposed by the British Nurses' Association to be attempted, a serious blow would be dealt at that steady progress which has marked the position of nurses in recent years, and that the step contemplated is one which, if taken, would leave most injurious consequences."

### A First Experience.

In Two Parts.—Part II.

We day nurses always breakfasted at 6.30, came on duty at seven o'clock; at nine we were allowed a quarter of an hour in which to make our own beds, tidy our rooms and ourselves after the sweeping, bed-making, dusting, etc., in the wards.

The nurses went to their dinner at 12.30, and we probationers at one. We had tea in the wards, which we made ourselves, at four o'clock; we had two hours off duty every day, except Sunday, either in the morning or afternoon, as the sister saw fit. We were supposed to go for a walk then, but I was generally too tired, and used often to lie down on my bed and go to sleep. We each had a ticket with our numbers on it, which we gave up to the porter on going out of the gate of the hospital, and received again on entering. He put down in a book the time that each nurse went out, and when she returned. This book was examined each day by the matron, who by this means could tell to a minute how long each nurse had been out, and knew exactly if anyone was late, and reprimanded them accordingly.

The charge nurses had one whole Sunday once a month off duty, as well as one half-day once a month. The probationers had one whole week-day, from ten till ten, a month, but not the half-day; they also had a fortnight's holiday once a year at whatever time the matron chose. The paying probationers were not obliged to go on night duty, in all other respects they were treated in exactly the same manner as the paid nurses.

When I had been in this ward five weeks I caught scarlet fever; as soon as it was found out what was the matter with me I was wrapped in blankets, put into an ambulance carriage and taken off to the London Fever Hospital, where I remained seven weeks.

When I returned to my work I was put into a woman's surgical and accident ward, the sister of which had a kind heart, but a very short temper. (Hospital tempers are short, I suppose because everyone has rather more work than they can possibly accomplish.) The one thing that upset her more than anything else was to have her probationers changed, which they were every six or seven weeks. Then everyone had a bad time; everybody was scolded—nurses, wardmaid, patients; nobody escaped, and, of course, the poor probationer worst of all. The first week in that ward my life was a burden to me, and it was only my pride that kept me from throwing it all up and going home by the first train. Everything that I did, or did not do, I was scolded for. A thing I had been told to do one day, I was blown up for doing the next. When the surgeons were in the wards, I was sent to fetch some dressing or appliance that I had never heard of before, and when I was frantically looking in every cupboard and every drawer, in the vain hope that I should know by instinct what it was like, sister would come thundering down the ward, shaking the hospital to its very foundations, her dress streaming behind her in the wind, fire and fury in her eye, calling upon Heaven to know what she was to do with such a fool! I often thought she was going to box my ears, but she never did. After the first week we got on better, she found out I was not quite an idiot, and in a little while she used to praise me, and say she would sooner have me under her when there was an operation to be done than any of her other probationers, and on Sunday, when one of the charge nurses had her day off, I always took her ward. Sunday was my hardest day. We always went to church morning or evening as sister decided. I used to get on very well till the evening, and then I used to get almost frantic; the wards had to be ready for the night by half-past six, as then a Scripture-reader used to come and give a service, and no one must move while he was there. The patients were allowed to see their friends from three till five on Sunday, and after they had gone the teas were given. That and the washing up of the tea-things took very nearly an hour; then there were the temperatures to take; and, for some unexplained reason, the sheets were changed on a Sunday night. There were never quite sheets enough to go round, and every nurse tried to be beforehand with the others, so as to get her full number. Sometimes they very nearly came to blows. To change twelve or fourteen pairs of sheets single-handed is no light task, even when you have plenty of time to do it in; but when half-an-hour is the utmost you are allowed, and some of the patients are so helpless they cannot raise themselves in bed, it is almost impossible. Sometimes a probationer from another ward, if the charge nurse was particularly amiable, would be sent to help me for a few minutes, but generally she was too busy in her own ward. Added to this, there were dressings to be done, as on a Sunday there are no students, and the nurses do the dressings.

My readers must not think that hospital life is all hardship and scoldings. There is a great deal of happiness mixed up with it. I got very fond of some of the sisters and nurses, and many of the patients were very nice and so grateful for everything that was done for them, and all those that possibly could used to help to their utmost with the ward work. I used to be quite sorry when they got well and were discharged, knowing that in all probability I should never see or hear anything more of them. I liked the surgical and accident wards best, for in these the patients are often not really ill or diseased as they are in a medical ward, and when they are not in pain they are often quite merry and cheerful; it used to be very difficult sometimes to keep them from laughing and talking too much and disturbing those who were very ill; they were allowed to sing from six to seven in the evening, and they used to count the minutes till six o'clock. Some of them used to sing very well, only with a dreadful cockney accent, which, however, I soon got used to.

I use to like preparing the dressings and getting everything ready for the surgeons, and when I got to know the names of the different things and where they were kept, and knew when they were likely to be wanted, I used to like going round with them, helping them and seeing how the different dressings were done. Sometimes a clumsy student would provoke me dreadfully, and I used to long to take the forceps or scissors out of his hand, and do his work for him. Then how I used to enjoy my holidays. It was worth while working hard—it made you appreciate them so. I shall never forget my first day off. What a pleasure it was putting on a fashionable dress and bonnet, and to know that for twelve long hours you could do just as you liked, without any fear of breaking rules or being scolded. How delightful your friends' society was; how delightful it was to feel it was really a pleasure to somebody to have you with them! It was a bright autumn day, and the Kensington Gardens looked so pretty with the sun shining on the Albert Memorial in the foreground, and a blue haze seen through the trees in the distance, all the more beautiful because my eyes had been restricted to the dingy surroundings of the hospital for so long. Yes, though a nurse's life is hard, there are many compensations. Surely it is worth anything to feel that you are of use to someone, and that you are helping to make somebody's heavy burden a little easier to be borne.

### Presented.

"I allus expected it."

"It wor a mussy she wasn't killed outright. Still, I dunno, it might ha' bin better than lingerin' on in the 'orsepital. Poor Margy! She couldn't help her nature, in course, but she was allus dream, dream, dreaming, so it ain't much of a surprise that she should be run over."

The speaker was right. It was not surprising that Margy lay in the hospital grievously injured. The dreamy slip of a girl belongied to nobody, but had been everybody's slavey, and was always wandering about as if her wits were out holiday-making. Knocked about from her cradle—a cold doorstep—the homeless orphan had tried to grow up in the wretched slum where her finder had taken her. But Margy had not managed the growing-up very well; she was stunted and white, and did not make much way, with the exception of her great blue eyes, which seemed to increase out of all proportion to the tiny face. Very beautiful would these eyes have been but for a certain mistiness about them as if an almost invisible veil were between them and the objects on which they rested.

"There's not the slightest hope," pronounced the doctors after examining the crushed, frail body brought into the hospital from under the horses' feet. "Give her everything she asks for; it will be an affair of a few days, and the end will come suddenly, at the last."

But they were not quite correct. The few days became weeks; then a month.

"Most singular vitality!" and the doctors marvelled. But then, some human beings have that strange tenacity of life, while others slip down "the narrow stream," all too easily, and seemingly regardless of the bitter anguish of those who would hold them back.

"Tell you how it happened, ma'am?" Margy lay back with her great blue eyes fixed on the tops of the Park trees, which she could see out of the window. Her arms rested outside of the coverlet, they were uninjured, the fatal mischief being internal. In one wasted hand she held a white rose; occasionally she lifted it to her lips, and softly issed it. Beside the bed sat the Sister who had tenderly spoken, day by day, to the dying girl of that land to which she was hastening. Margy had swiftly grown to love the sweet, compassionate face, and daily watched wistfully for its owner's coming. "Well, ma'am," went on the sufferer. "You see they were talking in our court about the great doings in the West End; the folk said there was to be a drawin'-room, where beautiful ladies went to make their curtseys to the Queen. I said I'd like to go and curtsey, too, and they all shouted at me. But I went, next day, to see the ladies. And I saw them; there was one that I thought must be a real angel. For a moment I got close to the carriage, and looked right in at her. Her hair was like real gold, and her face so soft and kind it made me feel like crying. And, ma'am, she'd such a posy! Hundreds of white roses it seemed; and all about her dress lay white roses, and in her hair somethin' like water was shinin'. Then someone pushed me, and I fell under the horses' feet just as the carriage began to move—and now I'm here," she ended, and the Sister sighed.

A pause followed. Margy watched a baby cloud sail across the blue over the tree tops, and faintly wished the window were wider that she might see where it went to. Strange thoughts and imaginings hovered about Margy's brain in those days. Her gaze was lifted up above the things of this world, and her eyes would fain have pierced the blue to see those streets of shining gold, those gates of pearl of which the Sister had told her. All the ugliness of the dreamy girl's life had dropped out of her memory; all the grinding poverty; all the squalor of the City alley. How easily all things do slip from us when the face of the King of Terrors approaches! The seeming tight grasp of earth is loosened in the hour—almost in the moment—of some great peril or calamity that crushes us without warning. Between Margy's soul and the body on the hospital bed there was a struggle—soon to be ended.

"Sister," began Margy, lifting the white rose to her lips, "is it only ladies who curtsey before the Queen? Wouldn't she like me to bow to her?"

"Yes, dear child, our good Queen cares for the poorest of her people; but the curtseying you are thinking so much about is only for ladies of rank and wealth who can go to Court in beautiful clothes and jewels."

"Is that all? Then, Sister, when I get my beautiful white robe and the crown of gold that you say is all ready for me up there, they will let me go to the Queen's drawin'-room, won't they? And when I curtsey the Queen will smile at me, and say, 'Why, that's poor Margy, who used to be so shabby.'"

The Sister turned to gaze at the child. Could she be delirious or—? But Margy was not wandering. Her eyes suddenly grown strangely clear, were fixed steadily on the blue. It was getting very near the end of the little pilgrim's journey.

"Little one!" The Sister knelt beside the white bed, and took the hand that had not the white rose. "The white robe and the crown are indeed ready, but you are to wear them in the presence of the King of Kings, your Heavenly Father, which will be far better. And, my child, there you will find perfect joy, perfect peace."

A strange, grey look had crept over the little face, the laboured breathing became quicker and shorter.

"Far better!" panted the sufferer; "my white robe and my crown of gold—and, oh, Sister, don't forget some white roses—" and in a moment Margy had slipped away to adorn herself for the presentation to her Heavenly Sovereign. The soul so full of passionate love for all beauty had gone where it would be steeped in the same.

By-and-by, when the Sister tenderly placed white roses about the slight young form, her tears fell on the pure flowers, and she told herself that to poor Margy her sorrowful, dreary life on earth was already being made up for a thousand-fold.

### Everybody's Opinion.

*[Correspondence on all subjects is invited, but we cannot in any way be responsible for the opinions expressed by our correspondents. No communications can be entertained if the name and address of the correspondent is not given, or unless one side of the paper only be written on.]*

### A PLEA FOR NURSES.

Greta writes: I know you must have very little space to spare in your valuable paper, but I will be much obliged if you will kindly insert the following note: 1st. Why are nurses allowed so few, indeed I may say with few exceptions, no opportunities of receiving Holy Communion at an early service? I know of several hospitals where the nurses are not obliged to go on duty before 8 a.m., and many of them would gladly give up some of their time for sleep to go to an early celebration so as to be back in time to go on duty at the usual hour. If matrons would only interest themselves more in their nurses, they could see that those who wish to worship and serve their Master make the kindest and most conscientious nurses, and are more self-denying. It does cost a tired nurse some little effort to give up an hour of her well-earned rest, but speaking from experience, I know what a comfort and help these early services are, and instead of tiring us, as perhaps the matrons fear, only strengthen us for the often hard and trying day we have to spend in the wards. The other point to which I wish to draw attention is the irregularity of th off-duty hours given to nurses. Surely it would be easy enough to have a fixed time for all the nursing staff, so that unless anything very abnormal occurred the nurses could be quite sure, from day to day, what hours they would be off duty, and thus be able to make appointments to meet their friends; for a nurse's time off duty is so short that her only chance of seeing her friends is to ask them to meet her when she is able to go out, and how very trying it is to a nurse's feelings when she has been longing and watching for the hour when she may reasonably expect to go out to be told "Oh, you cannot go out now, you must take such a time instead." She knows that her friends will wait for her in vain. And why is this change in the off-duty time often made? (I am alluding now to the smaller hospitals, for I know in large ones things are more regular). Only because the charge-nurse or ward sister wishes to go out then, and easily persuades the matron that it can make no difference to the probationer what time she goes. If matrons and ward sisters would only try a little more than they do to enter into their probationers' feelings they would be amply repaid by the love and devotion they would gain, but when matrons keep themselves on a pedestal, and leave the junior nurses entirely to those who are only looking out for themselves, they are the cause of turning many a warm-hearted girl into a bitter, callous woman, who will one day treat those under her as she herself has been treated. A gentleman once remarked to me that he often wondered why most nurses had such a hard expression. Is not this the result of their training? If a girl goes fresh from home and enters a hospital where no one shows any feeling for her or seems to take any interest in her, but she is treated as a mere machine, if she does not, in the course of time, become more or less callous and hardened she must be one of the wonders of the world. I do not wish to say that all matrons and ward sisters take no kind interest in their nurses. I have known and worked with some who, I am glad to say, are always ready to help their nurses in any way they can. But I am sure many nurses will agree with me that I have only stated what, in many cases, is the truth.

### THE REGISTRATION OF NURSES.

A Hospital Secretary writes: I desire to point out certain evident misapprehensions which frequently prevail as to the present position of the proposed scheme for the registration of nurses. "Were the scheme of Registration proposed by the British Nurses' Association to be attempted, a serious blow would be dealt at that steady progress which has marked the position of nurses in recent years, and the step contemplated is one which, if taken, would leave most injurious consequences." The Hospitals' Association, which originated the idea of registration for trained nurses, and which worked out a scheme with this object before the British Nurses' Association was even thought of, abandoned the idea, because they became convinced that a general register for nurses was rightly regarded, by the most capable of forming a sound judgment on the question, as being calculated to produce more evil results than good.

The above paragraph is taken from a pamphlet, by Miss Luckes, matron of the great London Hospital, published by Messrs. Churchill. Miss Luckes writes as the representative of "many experienced matrons, sisters, and nurses, who are distinctly of opinion that a result decidedly injurious to trained nurses, both individually and as a body, would be entailed if the suggested plan of registration were carried out." You could not spare space to give the detailed grounds of objection which Miss Luckes urges in this weighty pamphlet of which I enclose a copy, but a word or two may be added to objection III. There can hardly be a greater mistake with regard to the needs of nurses than to imagine that convalescent homes—that institution life of any kind—would prove a refreshment to tired or sick nurses needing rest and change. Of all persons they require home life, or its equivalent with friends whose interests lie outside the comparatively narrow groove of hospital life. This is only a sample of several impracticable proposals which the active promoters of the British Nurses' Association have advanced as proof of their want of practical acquaintance with all that is best and most efficient in the nursing world of to-day.

I have merely brought this instance forward in order that the public may realise what the hospital and nursing world have decided after careful examination and due consideration—viz., that the registration scheme of the British Nurses' Association is not calculated to advance but to retard the growth of efficient nursing.

Besides, the idea that the public cannot protect itself from incompetent nurses, as matters stand at present, cannot be seriously entertained, when nearly all the metropolitan hospitals and very many large provincial hospitals keep highly trained private nursing staffs for the use of any citizen who is in need of their services.

### Appointments.

[It is requested that successful candidates will send a copy of their applications and testimonials, with date of election, to The Editor, The Lodge, Porchester-square, W.]

Hull Royal Infirmary.—Miss Esther H. Bodger, who for the last three years has acted as matron of the West Sussex Infirmary, has just been appointed lady superintendent of the Hull Royal Infirmary. Miss Bodger trained at Westminster, which she left to become assistant matron at Sussex County Hospital. After some experience in a cottage hospital, Miss Bodger became matron of the Brecon and Brecknock Infirmary. Her experience has therefore been very wide, and she takes considerable capabilities to the task of ruling at Hull.

Whitworth Hospital.—Miss Burford, late matron of the Salford Nurses' Home, has been appointed first matron of the Darley Dale Hospital, built by the Whitworth Trustees, and now nearly completed. The hospital is to hold twelve beds, and is well fitted with all modern appliances. Miss Burford trained at St. Mary's, and afterwards worked for the South London District Nursing Association. A memorial begging Miss Burford to remain at Salford, was signed by 40 of the leading doctors and clergy of Manchester.

Garston Accident Hospital.—Miss E. F. Hunt was elected matron of this institution on the 4th inst., and enters on her new duties on July 11. Miss Hunt trained at the York County Hospital in 1882, and took her certificate. She afterwards entered the Liverpool Northern Hospital, where she spent two years as head assistant, and gained much experience in both medical and surgical work. For the last three years Miss Hunt has acted as staff nurse at the Bootle Borough Hospital, where she has made many friends. The night before she left there she was entertained at supper by the matron, house surgeon, and nurses, and several nice presents were made to her as parting gifts.

### Meetings.

The first annual meeting of members of the British Nurses' Association will be held at Cambridge on July 31st, at 11.45 a.m. Tickets, 4s. 8d. from London, and 2s. for the dinner, can be obtained on application to the Secretary.

### Vacancies.

The following vacancies are announced:

### Matrons.

Chichester Infirmary, £50.

Manchester Eye Hospital, £80.

Newcastle Royal Infirmary, £100.

North of England Children's Sanatorium, £50.

### Nurses.

Bedford Nurses' Institute, £26.

Birmingham Scarlet Fever Hospital (two), £20.

Blackheath Nursing Institution, £20.

County of Cornwall Nurses' Home.

Dumfries Royal Infirmary, £20.

Hampshire Nurses' Institute.

Harrogate Cottage Hospital.

Hollond Institution, Nice.

Ipswich Hospital, £25.

Kingston Home, Hull.

Northamptonshire Institution.

Nurses' Home, Harrogate.

Queen Victoria Nursing Institution, Wolverhampton.

Royal Infirmary, Windsor, £20.

Royal Surrey County Hospital, Guildford, £20.

St. Saviour's Infirmary, Dulwich, £20.

Surbiton Cottage Hospital, £25.

Victoria Cottage Hospital, Guernsey

Weston-super-Mare Nurses' Institute.

Workhouse Infirmary Nursing Association.

### District Nurses.

Bolton District Nurses' Home, £25.

Bristol District Nurses' Society.

Burton-on-Trent Institution, £28.

Wantage Cottage Hospital, £25.

### Probationers.

Bristol District Nurses' Society.

Bristol Royal Infirmary.

Convalescent Hospital, Bushey Heath, Herts.

Central Ophthalmic Hospital, W.C.

St. Helens Cottage Hospital, Lancs.

Truro Infirmary, £9.

Victoria Infirmary, Northwich.

### Notes and Queries.

To Correspondents.—1. Questions may be written on post-cards. 2. Advertisements in disguise are inadmissible. 3. In answering a query please quote the number. 4. If a private answer is desired, a stamped addressed envelope must be forwarded.

### Queries.

A mother asks where she can take her son who has recently recovered from an attack of scarlet fever, and who is therefore not yet fit to reside in lodgings.—Convalescents of this kind may be sent to Miss Mary Wardell's Convalescent Home, Great Stanmore, Herts (*Editor*).

(36) A doctor asks "the number of beds in the Buckinghamshire Infirmary, and whether it is well-managed."

(37) *Convalescent Home.*—Where is there a home which will take a boy with hip disease and abscesses for six months? *Kate.*

(38) *Nursing in New York.*—I should be glad to know the addresses of some private nursing institutions in New York.—*M. O.*

### Answers.

(30) *Hollond Institution.*—As a certified nurse who has nursed for this institution under the present directress, Miss Woodcock, I cannot say too much in its praise. In my long and varied experience, I have never met with so much kindness, sympathy, and comfort as there. The directress, who is much loved by her nurses, spares no pains to make them happy and contented. Should "Parish Nurse" be fortunate enough to get an appointment there, she will find the Hollond Institution a home in more than name. I shall be happy to give "Parish Nurse" any further information, if she will furnish me with her address. *K.*

(36) The number of beds is 50 (see "Burdett's Hospital Annual, 1889"), and this infirmary was established in 1833.

### A First Operation.

"The courage of the nurse—that courage which she will surely be called upon to exercise—is a moral one more than a physical courage. We all require it, no matter what path in life we follow. How well I remember the first test I received in this direction, when, a smooth-browed boy, I captured from a dispensary my first serious capital operation. Flushed with enthusiastic confidence, all was prepared in the dingy tenement where I was to operate. The patient, an old man, was being etherised in an adjoining room, when his wife, a burly Irishwoman, who seemed twice my size, called me aside, uttered her remonstrance against the operation, which she disapproved, and terminated her vigorous peroration by shaking her fist under my nose and saying: 'Well, you may operate, but if you kill him I'll have your life.' The memory of the cold shudder that ran down my back I can almost recall now, and it is, perhaps, needless to add that that patient received extraordinary care, and made a prompt recovery after his operation."—*Dr. E. L. Keyes.*

### NOTICE.

The Matron of the Princess Alice Memorial Hospital wishes to thank all the nurses who have responded to her advertisement, and to say that the vacancy has been filled up.

